# Social and Poor Law Problems

**Published:** 1907-04-06

**Authors:** 


### SOCIAL AND POOR LAW PROBLEMS.

### THE STEPNEY VISITORS' ASSOCIATION.

This Association has published its first triennial report. The Association was formed to join together groups of visitors attached to the different churches, missions, and settlements in the borough, with a view to spreading among the people with whom they came in contact sensible views on health, thrift, and such parts of the law as were most likely to affect their welfare—for example, the Factory Acts and the laws affecting tenant and lodger. The visitors were also to gather information as to special distress in the neighbourhood they frequented, cases of overcrowding, defective sanitary arrangements, and the like. The Chairman of the Society is the Bishop of Stepney, and the Association includes the pastors of most of the denominations in the neighbourhood, as well as Dr. D. L. Thomas, the Medical Officer of Health for the borough of Stepney, and some gentlemen connected with Toynbee Hall. A lady, Miss Forrester, was appointed Health Visitor, with instructions to give special attention to the subject of infant mortality. It was unfortunate that Miss Forrester's work should have aroused irritation, but so it was. She felt it her duty to report certain parents, who had given false addresses to the Registrar, with a view to avoiding the vaccination of their children, and owing partly to this circumstance it was thought wiser to transfer her from Stepney to a district in St. George's-in-the-East. Among other projects which the Association has in view, though it has not yet been able to carry them out, is the visiting of parents whose children are on the point of leaving school, to try to induce them to place their children in Friendly Societies, or adopt some other sound means of providing against sickness; and—and this is so important that one must deeply regret any delay in starting it—the visiting of hospital out-patients and recently discharged in-patients, to secure the regular and intelligent carrying out of the doctor's instructions. The Association has a phthisis sub-committee, which arranges lectures for health visitors who wish to know the best means to combat the disease, and seeks to learn the names of phthisical patients who are willing to accept visits and advice. The work done by the Association, although as yet largely tentative, is so important that it is a pity that it should be hampered as it is by lack of funds. An anonymous donor has contributed the money required for the Health Visitor's salary, but the Association has to depend upon voluntary work for a Secretary, and naturally it is difficult to find anyone who can give the necessary time to the work without remuneration, while hitherto the office expenses have been met by grants from the Bishop of Stepney, and by affiliation fees of half-a-crown from each of the agencies affiliated with the Association. These form a very inadequate income for the performance of such important work—work which, because it is preventive rather than remedial, is comparatively invisible, but none the less important.

### "CUTTING OFF THE WATER."

The practice of cutting off the water-supply in cases where the water-rate had not been paid was common when the water-supply of London was in the hands of private companies, and on the simple economic principle of preventing a customer having what he could not or would not pay for, nothing can be said against it. The water companies meant to make a profit, and acted as the law permitted them to do in pursuit of that object. But now that the supply is in the hands of the Metropolitan Water Board, another point of view has come up—the question, namely, of the effect on public health of a scanty supply of water. Bearing in mind the risks attendant on the want of water, the Borough Council of Islington last year addressed a communication to the other Borough Councils in the Metropolis, requesting each to ask its representative on the Metropolitan Water Board to "urge upon the Board the discontinuance of the practice of cutting off the water-supply from premises in respect of which the water-rate had not been paid, and to substitute legal proceedings for the recovery of such rate when necessary." The Board has not consented to do this, but in a communication received by the Councils from the Clerk of the Water Board, it is mentioned that the subject had been very carefully gone into by the Finance Committee in February 1905, and that the Committee do not think that it would be in the interests of the Board, and, therefore, of the general body of consumers, to abandon entirely the practice of disconnecting the water-supplies of defaulters in the matter of payment. The appeal of the Councils has, however, had some effect, for the Clerk goes on to say that since the memorial had been received the power to cut off supplies had been very sparingly used, and the number of disconnections had been reduced to a minimum. No supplies are disconnected without the order of the Committee being first obtained, and in each case the whole of the surrounding circumstances are brought before them and fully considered. It has been the practice to refrain from giving such orders when the premises are known to be occupied by more than one family, and in genuine cases of poverty. In cases where the owner of the property, and not the occupier, is liable for the payment of the water-rate, either by law or by agreement, the Board is precluded by the Water Companies (Regulation of Powers) Act of 1887, from cutting off the water-supply. How sparingly the powers of disconnecting supplies is used by the Board may be inferred from the fact that in 1906, out of a total of nearly 1,100,000 supplies, only 213 were actually withdrawn, and of these, 132 were merely turned off at the stop-cock, leaving only 81 cases in which physical disconnection was made. As the Board's area of supply comprises 252 parishes, it is evident that there is no great harshness shown. But, as Dr. Dudfield, the Medical Officer of Health for Kensington, says, after quoting this statement in his report, it is doubtful whether it can really be worth the while of the Water Board to exercise their power of cutting off supplies for non-payment of rate, and whether it would not be better to remove all ground for adverse criticism by discontinuing the practice and taking legal proceedings against the small number of rate defaulters.

Of the many puzzling cases that come under the medical man's notice none, perhaps, surpass in interest and in obscurity those which are lumped, for convenience sake, under the generic name of "osteoarthritis of the spine." The anomalous symptoms which this condition gives rise to, has led to it being described as so many separate diseases. Synonymous with spinal ankylosis from ill-understood causes are no fewer than fourteen clinical conditions to which different authors have given different names. In the March number of the *Glasgow Medical Journal*, Hunter attempts to collate the 200 odd reported cases. He regards the disease as a rheumatoid arthritis, affecting almost entirely the vertebral joints. The generalised muscular atrophy, the fibrillary response to mechanical stimuli, the contracture of the muscles, the thin, pigmented skin, and the ankylosis of joints, he remarks, are highly suggestive of rheumatoid arthritis. According to him, all the recorded cases fall into one or other of four groups. The first group include all cases of undoubted rheumatoid arthritis; the second, those in which there is a definite history of rheumatic fever; the third cases in which trauma is probably a causative factor; while in the fourth group he would put the ankylosis of elderly people whose occupation entails a persistent stoop. He thinks most of these cases are due to a circulating toxin with a widespread action on certain trophic centres, and lays stress on the importance of a careful and exhaustive examination, not only of the joints, but of the cord and peripheral nerves, in cases that are available for post-mortem examination.

